# Quantitative Chest CT Analysis to Measure Short-Term Sequelae of COVID-19 Pneumonia: A Monocentric Prospective Study

**DOI:** 10.3390/tomography8030130

**Published:** 2022-06-17

**Authors:** Ezio Lanza, Angela Ammirabile, Maddalena Casana, Daria Pocaterra, Federica Maria Pilar Tordato, Benedetta Varisco, Costanza Lisi, Gaia Messana, Luca Balzarini, Paola Morelli

**Affiliations:** 1 Department of Diagnostic and Interventional Radiology, IRCCS Humanitas Research Hospital, Via Manzoni 56, 20089 Rozzano, Milan, Italy; ezio.lanza@humanitas.it (E.L.); costanza.lisi@humanitas.it (C.L.); luca.balzarini@humanitas.it (L.B.); 2 Department of Biomedical Sciences, Humanitas University, Via Rita Levi Montalcini 4, 20090 Pieve Emanuele, Milan, Italy; benedetta.varisco@gmail.com (B.V.); gaia.messana@gmail.com (G.M.); 3 Department of Infectious Diseases, IRCCS Humanitas Research Hospital, Via Manzoni 56, 20089 Rozzano, Milan, Italy; maddalena.casana@humanitas.it (M.C.); daria.pocaterra@humanitas.it (D.P.); federica_maria.tordato@humanitas.it (F.M.P.T.); paola.morelli@humanitas.it (P.M.)

**Keywords:** COVID-19, lung diseases, computed tomography, quantitative CT

## Abstract

(1) Background: Quantitative CT analysis (QCT) has demonstrated promising results in the prognosis prediction of patients affected by COVID-19. We implemented QCT not only at diagnosis but also at short-term follow-up, pairing it with a clinical examination in search of a correlation between residual respiratory symptoms and abnormal QCT results. (2) Methods: In this prospective monocentric trial performed during the “first wave” of the Italian pandemic, i.e., from March to May 2020, we aimed to test the relationship between %deltaCL (variation of %CL-compromised lung volume) and variations of symptoms-dyspnea, cough and chest pain-at follow-up clinical assessment after hospitalization. (3) Results: 282 patients (95 females, 34%) with a median age of 60 years (IQR, 51–69) were included. We reported a correlation between changing lung abnormalities measured by QCT, and residual symptoms at short-term follow up after COVID-19 pneumonia. Independently from age, a low percentage of surviving patients (1–4%) may present residual respiratory symptoms at approximately two months after discharge. QCT was able to quantify the extent of residual lung damage underlying such symptoms, as the reduction of both %PAL (poorly aerated lung) and %CL volumes was correlated to their disappearance. (4) Conclusions QCT may be used as an objective metric for the measurement of COVID-19 sequelae.

## 1. Introduction

Since December 2019, the spread of Coronavirus Disease-19 (COVID-19) rapidly increased worldwide, being declared as a pandemic on 11 March 2020. [1].

In the acute phase, COVID-19 manifests mainly with fever, cough and dyspnea that could need a variable degree of respiratory support, from non-invasive ventilation to intubation and ICU admission. [2] Few data are available about the persistence of symptoms, especially in hospitalized patients with severe acute forms that appear to have a higher risk of pulmonary sequelae [3,4,5].

The role of Computed Tomography (CT) in the COVID-19 detection has been early investigated; this imaging technique demonstrated high sensitivity in pneumonia diagnosis, also in patients with negative reverse transcriptase-polymerase chain reaction (RT-PCR) [6]. Bilateral ground-glass opacities with peripheral localization have been reported as the typical CT findings, especially in the initial stages of the disease [7,8]. Quantitative analysis of CT scan has demonstrated promising results in the prognosis prediction, using segmentation algorithms and extraction of lung volumes according to Hounsfield Unit (HU) intervals [9,10,11,12].

The scope of this prospective monocentric trial was to assess the evolution of respiratory symptoms at short-term follow-up in patients who survived the first wave of the Italian COVID-19 pandemic and to correlate them with the change of the appearance of lung parenchyma as measured by QCT.

## 2. Materials and Methods

### 2.1. Patient Population and Study Design

The Institutional Review Board (IRB) of Humanitas Clinical and Research Hospital approved this study. Each patient signed an informed consent agreeing to receive the proposed medical care.

This was a single-centre prospective study to assess the evolution pattern of COVID-19 pneumonia.

From March to May 2020, a period corresponding to the pandemic “first wave” in Italy, all patients hospitalized for COVID-19 pneumonia at our Institution received a chest CT upon acceptance to evaluate the disease severity according to an internal protocol approved by the IRB. Upon hospital discharge, all surviving patients were asked to participate in this follow-up study, involving (a) 30–60 days follow-up clinical assessment with a standard questionnaire regarding residual pneumonia symptoms (b) same day non-contrast chest CT.

Overall, inclusion criteria were as follows: (1) symptomatic COVID-19 with dyspnea, cough and chest pain, (2) microbiological confirmation of SARS-CoV-2 infection, (3) chest CT scan and complete laboratory tests at admission, (4) follow-up chest CT scan and simultaneous clinical assessment after discharge, (5) recorded pharmacological therapy during hospitalization. Patients whose chest CT showed respiratory artefacts influencing image analysis were excluded from the study.

Full laboratory, clinical and radiological data were recorded, including patients’ demographics (age, sex, BMI, comorbidities) and hospitalization details (length of stay, type of pharmacological and oxygen therapy, complications). All the included patients were admitted in specialized clinical wards or in the intensive-care unit according to the disease severity; the time interval (days) from the onset of symptoms and the ER admission was also reported. The administered drugs were recorded either individually or in a generalized manner, i.e., antibiotics, anti-coagulants, antivirals or immunosuppressants.

### 2.2. CT Protocol and Image Analysis

Non-contrast chest CT examinations were acquired with a multidetector CT scanner (Philips Brilliance 64, Philips Healthcare, Amsterdam, Netherlands) with the following scanning parameters: tube voltage 120 kV, tube current 130–200 mAs, 240 mAs, collimation 64 × 0.25, pitch 1.4 mm. Reconstruction was performed with a slice thickness of 2.5 mm. Patients were scanned in the supine position with raised arms at full inspiration, if possible.

As previously described [13], the anonymised images were exported to a segmentation suite for medical image computing performing semi-automated segmentation (3D Slicer) [14], later validated or manually improved by experienced radiologists. Accurate lung segmentation included interstitial structures, segmentary vessels and bronchi and excluded the main pulmonary arteries and bronchi, pleural effusion, lung masses and mediastinal structures.

A quantitative analysis (QCT) of segmented structures was performed, according to the following criteria: depending on established ranges of Hounsfield Unit (HU), total lung volume was subdivided into hyperinflated from −1000 to −901 HU, normally aerated from −900 to −501 HU, poorly aerated (%PAL) from −500 to −101 HU, non-aerated (%NNL) from −100 to 100 HU. An additional key parameter namely compromised lung volume (%CL = %PAL + %NNL) was computed, as the sum of poorly aerated (%PAL) and non-aerated (%NNL) lung, which included pulmonary areas with a density ranging from −550 and 100 HU.

The differences between %CL and %PAL values obtained between admission and follow-up CT scan were calculated for each patient (i.e., %deltaCL, %deltaPAL, with %delta = 100 (follow-up–admission)/admission).

### 2.3. Outcome

The aim of our study was to test the relationship between %deltaCL and variations of symptoms-dyspnea, cough and chest pain-at follow-up clinical assessment after hospitalization.

### 2.4. Statistical Analysis

Descriptive statistics were carried out using frequencies and percentages for categorical variables and median and IQR for quantitative variables. We performed binomial logistic regression analyses, including residual symptoms of cough, dyspnea and thoracalgia as dependent variables, whereas potential predictors considered were: length of stay, cortisone administration, heparin administration, symptomatic days before hospitalization, oxygenation support, pre-existing lung pathology (smoking, COPD), diabetes, d-dimer > 250 ng/mL on the day of admission. The process of variable selection was carried out using both stepwise forward and backward methods of entry. Key variables locked in the respective analyses were %deltaCL and %deltaPAL in six different analyses. A cut-off *p* < 0.2 was considered for inclusion in the final model; *p* < 0.05 were considered significant.

STATA16 (StataCorp LP, College Station, TX, USA) was used for all analyses.

## 3. Results

During the considered time interval, 510 patients were admitted for COVID-19 pneumonia at our Institution; 390 (76%) survived hospitalization. Of these, 336 agreed to participate in the study and 282 (95 females, 34%) with a median age of 60 years (IQR, 51–69) were ultimately included for having complete clinical and radiological data (Table 1).

Median hospitalization was 9 days (IQR 7–15); 93 (33%) patients were discharged in 7 days, 206 (73%) before 14 days and 76 (27%) stayed more than 14 days.

Overall, 172 (61%) patients required oxygenation support; 15 (5%) were intubated; 110 (39%) patients maintained sufficient spontaneous oxygenation during observation and were not assisted with external oxygen. Thirty-nine (14%) had a pre-existing lung condition (smoking, COPD); 48 (17%) were diabetic.

All patients received the best supportive care considering the available evidence at the time of this study; of note, 107 (38%) were treated with subcutaneous heparin at a sufficient dose to achieve an anticoagulant effect; 21 (7%) received corticosteroids.

Upon admission, all patients received non-contrast chest CT. After QCT of lung disease, median %CL was 11% (IQR 6–18%) of which median %PAL, representing ground-glass opacities, was 9% (IQR 5–14%).

235 patients (83%) showed elevated d-dimer as measured within 24 h from admission.

The median time interval between admission and follow-up visit including repeat CT scan was 48 days (IQR 42–59). At control, 6 (2%) patients lamented residual dyspnea, 13 (4%) reported generalized chest pain and 3 (1%) had residual uncontrollable coughing.

**Figure 1:**
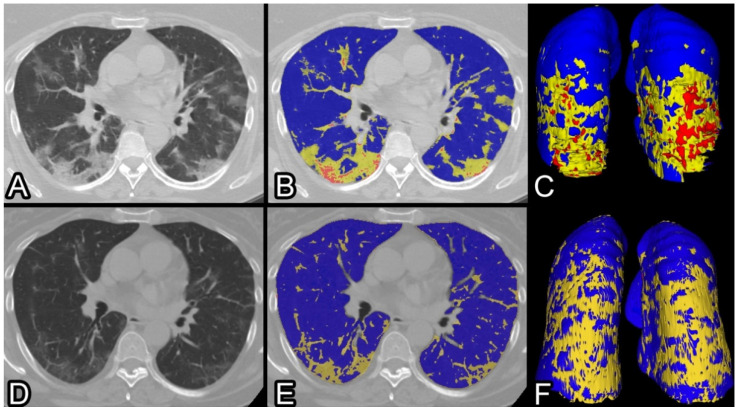
CT scans of a 57 years old lady affected by COVID-19 pneumonia, treated with non-invasive oxygenation during 11 days of hospitalization. At admission, she lamented dyspnea at rest, cough, thoracalgia and fever. (**A**) Non-contrast CT scan ad admission showing typical bilateral ground-glass consolidation (**B**) QCT of the same scan highlighting in yellow the poorly aerated and in red the non-aerated lung volumes (**C**) three-dimensional view of the whole lungs. After 44 days, a CT scan shows significant improvement (**D**) with subtle residual ground-glass opacities and linear scarring, better outlined at QCT (**E**) and using 3D reconstruction (**F**). At the follow-up clinical assessment, the patient lamented persisting laboured breathing and abrupt intermittent coughing. Residual %CL and %PAL were 6% and 5%, consequently %deltaCL and −%deltaPAL −12% and −10%.

Median follow-up QCT parameters were: 5 %CL (IQR 4–6%), 4 %PAL (IQR 3–5%); the median differences were: %deltaCL 5% (IQR −11/−1%), %deltaPAL 3% (IQR −9/−1%). (Figure 1).

After binomial logistic regression analyses, the following variables were significant predictors of residual dyspnea: %deltaCL (OR 0.82, *p* = 0.03) and %deltaPAL (OR 0.81, *p* = 0.02); thoracalgia was correlated with %deltaPAL (OR = 1.11, *p* = 0.02) and length of hospitalization, either in the model with %delta CL (OR = 0.85, *p* = 0.04) and %deltaPAL (OR = 0.84, *p* = 0.03); persistent coughing was associated with increasing age (OR 1.11, *p* = 0.04). Results are detailed in Table 2.

Additionally, %deltaCL and %deltaPAL were significantly better in patients who were hospitalized for more than 7 days (*t*-test, *p* < 0.001, LOS < 7 days vs. LOS > 7 days).

## 4. Discussion

We reported a correlation between changing lung abnormalities measured by QCT, and residual symptoms at short-term follow up after COVID-19 pneumonia. Our results highlighted how, independently from age, a low percentage of surviving patients (1–4%) may present residual respiratory symptoms at approximately two months after discharge. Of these, dyspnea was significantly correlated to the changes in lung impairment, as measured by the use of QCT. Specifically, a greater restoration of the normal lung architecture greatly lowered the risk of residual dyspnea. Length of hospital stay influenced residual thoracalgia, whereas age was the only significant predictor of residual coughing. No role could be highlighted for abnormal d-dimer documented at admission.

CT has played a central role in the diagnosis of COVID-19 for the presence of lung parenchymal abnormalities as signs of interstitial pneumonia, mainly bilateral GGOs with a subpleural distribution [15,16]. In our analysis, we focused on the %PAL, representing ground-glass opacities and %CL representing overall lung impairment, considering the high frequency of these opacities and their persistence after the acute phase that can correlate with altered respiratory function [17,18].

Our study proved that precise quantification of lung abnormalities and their evolution over time can correlate to clinical data; this relationship has been extensively demonstrated through the use of CT scores in the early phases of the disease [19,20,21]. In particular, the application of quantitative CT parameters has shown advantages over semi-quantitative visual evaluation in terms of accuracy [22]. Apart from diagnostic purposes, QCT can have a prognostic value, for example in the prediction of adverse outcomes during hospitalization, such as the need for supplemental oxygen, ICU admission or in-hospital mortality [13,23,24,25,26]. Considering the advantages of QCT and the dynamic evolution of COVID-19 on chest CT, temporal changes of COVID-19 features could be objectively monitored with serial CT scans and related to clinical types of the disease [27,28,29,30]. To date, studies have focused mainly on a short-term follow-up to assess imaging findings after the acute phase, generally after 14 days, when gradual resorption of lesions begins and appears to be slower in severe cases [31,32,33].

To date, little is known about the long-term outcomes of COVID-19, mainly about sequelae of severe acute forms. The definitions “Post-COVID syndrome” or “Long-COVID” have been used to describe the persistence of symptoms more than three weeks from the diagnosis with an estimated incidence in the range 10–35% [34]. Respiratory manifestations have been included in the list of the most common symptoms, especially in patients hospitalized during the acute phase of the disease, and their frequencies are highly variable [35,36,37]. We found residual symptoms persistence in approximately 7% of patients, a lower percentage compared to studies performed in the early convalescent phase. Two trials with a mean follow-up of 12 weeks after initial symptoms described persistent dyspnea in 20–29.5% of patients vs. 2% of our study. This difference could be explained by a much lower percentage of severe cases included in our study, with only 61% of patients requiring oxygenation vs. 100% and 76.7% in the aforementioned trials, and a lower degree of lung impairment [38,39]. Instead, another prospective study with a 4 months follow-up found residual dyspnea in 5.5%, chest pain in 0.4% and cough in 2.5%, more in line with our results. Their cohort consisted of 238 patients, mainly non-severe cases (70.6%), with a comparable distribution of oxygenation needs (27.7% no supplemental oxygen, 63.5% oxygen support and 8.8% mechanical ventilation) [40].

We are aware of some limitations of our study. First, further studies are needed in a larger cohort of patients and with a longer follow-up to make our results more representative. Then, during the first wave of the COVID-19 pandemic, there was not an established treatment regimen; these therapeutic differences could have influenced the prognosis and the outcome of the disease because therapy was generally carried out in patients with severe infection, prone to have major complications. Finally, our patients did not receive pulmonary function tests during the follow-up assessment, thus we cannot draw reliable conclusions regarding the impact of medical therapy.

In conclusion, we reported how a small percentage of patients surviving COVID-19 pneumonia develop residual respiratory symptoms at short term follow-up. QCT is able to quantify the extent of residual lung damage underlying such symptoms, as the reduction of both %PAL and %CL volumes is correlated to their disappearance. QCT may be used as an objective tool for confirmation of persisting lung injury after infection and indicate the need for prolonged medical therapy.

## Figures and Tables

**Table 1:** Patients’ demographics and clinical data.

Data	Median (IQR) or Number of Observations (%)
Age	60 (51, 69)
Females	95 (34%)
Males	187 (66%)
symptoms onset (days)	7 (4, 10)
%CL admission	11% (6, 18)
%PAL admission	9% (5, 14)
%CL follow-up	5% (4, 6)
%PAL follow-up	4% (3, 5)
%deltaCL	−4% (−11, −1)
%deltaPAL	−3% (−9, −1)
length of stay	9 (7, 15)
lung disease	39 (13%)
**Therapy**	
corticosteroid	21 (7%)
heparin	107 (38%)
oxygen	172 (61%)
intubation	15 (5%)
**Follow-up**	
thoracalgia	14 (5%)
dyspnea	6 (2%)
coughing	3 (1%)
days between scans	48 (42, 59)
days to negative swab	33 (25, 43)

IQR: interquartile range; %CL: compromised lung; %PAL: poorly aerated lung.

**Table 2:** Results from logistic regression analyses.

	OR	SE	*p*-Value	95% C.I.	
**Dyspnea**					
%deltaCL	0.82	0.07	0.03 *	0.69	0.98
Corticosteroid	8.50	8.38	0.03 *	1.23	58.63
Lung disease	3.60	3.54	0.19	0.52	24.77
Oxygen therapy	5.49	6.35	0.14	0.57	53.01
*LR chi2(4) = 12.54*					
%deltaPAL	0.81	0.07	0.02 *	0.67	0.97
Corticosteroid	7.47	7.06	0.03 *	1.17	47.63
Oxygen therapy	6.38	7.26	0.10	0.69	59.38
*LR chi2(3) = 11.25*					
**Thoracalgia**					
%deltaCL	1.05	0.03	0.08	0.99	1.11
Length of stay	0.85	0.07	0.04 *	0.73	0.99
Intubation	10.47	17.42	0.16	0.40	273.05
Age	0.95	0.02	0.06	0.90	1.00
Diabetes	2.83	2.13	0.17	0.65	12.34
*LR chi2(5) = 15.87*					
%deltaPAL	1.11	0.05	0.02 *	1.02	1.20
Age	0.95	0.02	0.05 *	0.90	1.00
Intubation	10.63	16.75	0.13	0.48	233.21
Length of stay	0.84	0.07	0.03 *	0.72	0.98
Diabetes	2.92	2.20	0.16	0.67	12.81
*LR chi2(5) = 18.64*					
**Coughing**					
%deltaCL	0.82	0.11	0.12	0.63	1.05
Age	1.10	0.06	0.06	1.00	1.22
*LR chi2(2) = 9.25*					
%deltaPAL	0.77	0.11	0.08	0.58	1.04
Age	1.11	0.06	0.04 *	1.00	1.22
*LR chi2(2) = 9.56*					

* statistically significant; C.I. = confidence interval; LR = likelihood ratio; OR = odds ratio; SE = standard error.

## Data Availability

The authors declare that the data of this study are available from the corresponding author on reasonable request.
